# Case study of posts before and after a suicide on a Swedish internet forum

**DOI:** 10.1192/bjp.bp.114.154484

**Published:** 2015-12

**Authors:** Michael Westerlund, Gergö Hadlaczky, Danuta Wasserman

**Affiliations:** **Michael Westerlund**, PhD, National Centre for Suicide Research and Prevention of Mental Ill-Health (NASP), Karolinska Institutet, Stockholm and Department of Media Studies, Stockholm University, Stockholm, **Gergö Hadlaczky**, PhD, and **Danuta Wasserman**, MD, PhD, National Centre for Suicide Reserach and Prevention of Mental Ill-Health (NASP), Karolinska Instituetet, Stockholm, Sweden

## Abstract

Websites and discussion forums have become an important and sometimes controversial source of information on suicide. Using a case report, our aim was to examine the responses, attitudes and beliefs that were communicated on a forum before, during and after a suicide act. We undertook two related analyses: a qualitative investigation of the messages that were posted before the suicide and a combined qualitative–quantitative analysis of the messages posted during and after the suicide. Nearly half the posted messages before the suicide encouraged the victim to complete the suicidal act, and a surprising number of posts after the suicide expressed excitement, although around half of the posts considered the suicide to be tragic. It is of great importance to increase awareness of suicide signals and understanding about how to respond to individuals who communicate suicide intentions on different forums on the internet.

At 11.51 h on 11 October 2010, a 21-year-old man started a thread entitled ‘Hanging’ on the Swedish internet forum Flashback.^1^ In his first post, the thread starter (TS) wrote that he had decided to hang himself and to display the suicidal act on the forum. In the following hour, 13 different forum participants posted 30 comments in the thread. Some participants tried to give him psychological support and talk him out of his suicide plans, others did not believe him and called him a ‘troll’ (internet slang for a person who starts arguments or upsets people by posting messages in an online forum, chat room or blog with the deliberate intent of provoking a response), and still others even encouraged him to put his plans into action. Shortly after 13.00 h the TS posted the comment ‘alright let's do it’ and then hung himself as his suicide was streamed live on the internet using a webcam. Some of the forum participants tracked his internet protocol (IP) address and called the emergency services. When the police and ambulance arrived at his home at 14.05 h he was already dead. This tragic incident received much attention in the media and brought on a heated debate about internet behaviour and what responsibilities participants, moderators and website owners have when individuals communicate risky and, in this case, life-threatening issues. This case also raises important questions about the process of – and responses to – suicide communication in the era of global digital communication on the internet.

Suicide is one of the major causes of death in the world, leading to approximately 700 000 deaths per year.^2^ It is estimated that by the year 2020, this figure will have increased to 1.5 million.^3,4^ While the topic of suicide is still taboo and stigmatised,^5^ and its significance underrated in most contemporary societies and cultures,^6^ websites and discussion forums have become an important and sometimes controversial source of information on the subject.^7^ With the massive communication possibilities that the internet has brought, problematic subjects like suicide have become more accessible to individuals than ever before.^8,9^ Today, the internet has become the main platform for information and communication about suicide.^10^ Conversations and disclosures regarding suicide occur on a large variety of internet forums,^11–13^ and it has been debated if online communication about suicide primarily provides opportunities or poses a serious threat for those who communicate about suicide-related issues.^14–17^ Computer-mediated communication has made it possible for participants to be anonymous and, simultaneously, enter into a public space to discuss and share personal thoughts, feelings and experiences about this still ‘forbidden’ subject. In other words, elements from these personal and intimate suicide communications have spread from the private sphere to the internet and have become, to a large extent, public and mass mediated.^8^ As in the ‘real world’ the actions people take on the internet affect their own and other people's lives. A study by Harris *et al*^18^ shows that individuals at suicide-risk who frequently search for suicide-related material on the internet score high on nearly all measured suicide-risk variables, including suicidal behaviour, suicidal ideation, suicidal plans, living alone, lower education, unemployment and a history of psychiatric diagnosis. They also report less perceived social support from family and friends, compared with other online users. Suicide-related online users find open, anonymous, unmoderated and peer-to-peer internet forums to be supportive and useful for their perceived needs – a ‘place’ where they could find participants similar to themselves – and that communication with family, healthcare professionals and help sites is less satisfying.

Even though suicide and suicide attempts may be seen as a consequence of internal psychic conflicts, it is also clear that external factors play a significant role in the complex suicidal process.^19^ Many studies have shown that a large proportion of those who attempt suicide have communicated their suicidal intent and plans, directly or indirectly, to other people before the act.^20^ A review of studies on suicide communication showed that between a third and a half of individuals who die by suicide had directly communicated their suicidal intentions to people around them.^21^ If indirect communication is included, the proportion rises to 60–80%.^20^ Owen *et al* take the concept of suicide communication further and define it as a suicide communication event, which is a ‘ … set of circumstances in which a person expresses suicidal feelings, thoughts, intentions or plans, either directly or indirectly, in interaction with other people in their social environment’.^20^ The essential point is that suicide communication events are the most important and observable features in the suicide process.

Although not that common, there have been a number of cases in recent years where people have displayed their suicidal act online. For example, a teenage boy in Miami, USA, took his own life live on the internet in November 2008^22^ and a 42-year-old British citizen broadcast his hanging in March 2007, after communicating his intentions on a chat room.^23^ These occurrences follow some similar patterns: for example, that the suicidal individuals first communicate their intentions on a forum, blog or a chat, and that the other participants seriously doubt the authenticity of the suicide messages. The aim of this study was to examine how participants on an internet forum act and react faced with suicidal communication and while witnessing the suicidal act. We examined the responses, attitudes and beliefs that are communicated on the forum before, during and after the suicide act.

## Method

**Fig. 1:**
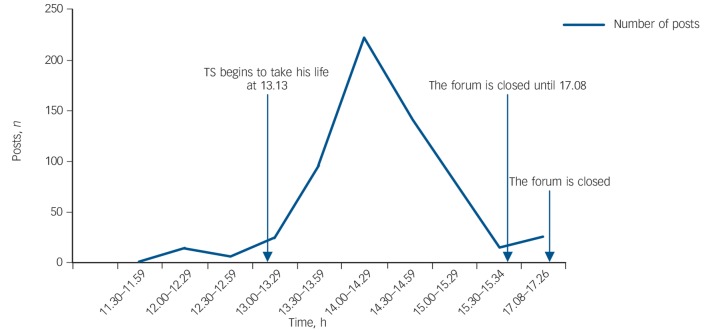
Number of posts in the communication thread over time. TS, Thread starter.

The material in the present case study consists of the 638 messages that were posted in the thread ‘Hanging’ on the Swedish internet forum Flashback^1^ on 11 October 2010, from 11.51 to 17.26 h, when the moderator closed the thread (Fig. 1, the thread was reopened in the afternoon the next day and another 3400 posts were written before it was finally closed down). Note that we considered it important for ethical reasons not to conduct or publish this study close in time to the suicide event (further details of ethical considerations are given in an online supplement to this paper). The case study includes two related analyses, a qualitative investigation of the messages that were posted before the TS's suicide and a combined qualitative–quantitative analysis of the messages posted during and after the suicide. Flashback (www.flashback.org) is a rather controversial internet discussion forum, with a history of lawsuits, furious debates and closures; established on the internet in 1995, it is one of the most popular Swedish websites with more than 900 000 members and over 50 million posts over the years. Its determined assertion of freedom of speech has also made it a home for information and discussions about highly subversive issues, such as extreme political views, information on illegal drugs, hacking, illegal downloading etc. The majority of visitors (90.4%) are located in Sweden and the forum has a small over-representation of male members.^24^ Practically all posts on the forum are written in Swedish. In this article the quoted examples from the thread have been translated from Swedish to English by the authors. The aim was that the translation should be as close as possible to the participants' language use. The thread ‘Hanging’ was started under the (static) subject headline/heading ‘Science and humanities’, with the subheadlines ‘Psychology and psychiatry’ and ‘Mental problems’.^1^ Because of the anonymous nature of the forum, it is difficult to determine specific characteristics of participants engaging in different threads. It is perhaps possible to assume that those who sought out this specific thread had a particular interest in issues related to mental ill health, possibly in parallel with mental problems on their own.

In the first qualitative analysis, the 30 posts^1^ before the suicide act are examined. In order to interpret the qualitative meaning of the text-based posts, the material was read in-depth by two of the authors (M.W. and G.H.) and sorted under different themes, in line with qualitative thematic analysis methods.^25^ Nine of the messages were posted by the TS and 13 were posted by other participants in the thread (some participants posted more than one message). The analysis is an attempt to understand what may be behind the TS's communication about his suicide plans and his choice to live-stream the hanging on the internet, as well as to describe how the participants in the thread responded to the TS's statement that he would take his own life, primarily if the suicide were discouraged or encouraged.

The material for the second qualitative–quantitative content analysis consists of the 608 messages^1^ that were posted during and after the TS's suicide, before the moderator closed the thread in the late afternoon of the same day. As in the first analysis, the material was first read in-depth by the authors and sorted under different themes,^25^ using a qualitative, inductive approach. In this first analytical step, certain responses, attitudes and beliefs from the participants concerning the TS's suicide in particular, and suicide from a more general perspective, were identified. The attitudes and beliefs found in the 608 posts regarding the suicide were then operationalised into quantifiable variables and categories, in line with a quantitative content analysis approach,^26^ and coded by one of the authors. An interrater reliability analysis was performed after a subset of the posts (10%) was coded by another author to determine consistency. The interrater reliability for the raters was found to be kappa (κ) = 0.67 (*P*<0.001), with 86% consistency. The analysed variables and categories are as follows (the figures in brackets are the number of postings that discuss each specific theme):

- Aspects of authenticity (*n* = 470):
  - believed that the TS did hang himself
  - did not believe that the TS died by suicide.
- Attitudes to the TS's suicide (*n* = 344):
  - tragic/horrible
  - interesting/funny
  - neutral.
- Opportunities for prevention (*n* = 95):
  - could/should have been prevented
  - could/should not have been prevented.
- Responsibility for the TS's suicide (*n* = 110):
  - other participants' fault
  - the TS's own fault
  - other reasons.
- Reasons for why the TS took his own life (*n* = 38)
  - mental illness/feeling bad
  - societal/social factors
  - stupidity.
- Perceptions of the images of the TS's suicide (*n* = 148)
  - horrible/tragic
  - cool/funny/exiting
  - neutral.

## Results

### Before the suicide

The thread ‘Hanging’ on the internet forum Flashback^1^ was started by the young man (the TS), declaring:

‘I have now decided to kill myself by hanging. I have softly tried to strangle myself and saw how that feels. Took some painkillers a few minutes ago (100 mg dexofen and 1500 mg paracetamol), now waiting for it to start working. Have turned on my webcam with a program that makes a screenshot every 2 seconds and put up an FTP [file transfer protocol, a standard way of transferring computer files on the internet] where the images will be available, will post the IP, port and login details before I do it.’

As can be seen in the initial message, the TS is straightforward and clear about his intentions, and very detailed about the live streaming of the hanging. He says that he prepared himself by previously testing how it feels to strangle himself and that he has taking pain relievers to reduce any possible pain. The first two replies the TS gets to this post is:

‘Good luck then!’

‘It can't be that bad … When everything is at its worst it can only get better …’

In the TS's second post, previous suicidal behaviours (i.e. self-strangulation) are described and discussed:

‘ … It has always been a scary thing to kill oneself, as you might understand … But after I tested strangling myself with my hands, so that the blood vessels in the face began to break, it did not seem so scary anymore, but more filled with peace, like I finally would come to rest.’

Of the 21 posted messages by other participants before the hanging, 7 can be characterised as encouraging, or inciting suicide, for example:

‘Stupid fuck, strangulation is no pleasure. Don't you have a car … carbon monoxide rules … ’

In another two posts, the authenticity of the TS's suicide plans is questioned, thereby provoking him to show that he is serious:

‘In the way you write, one can see that you're just a faker, go and hang yourself.’

Thus, almost half of the postings can be said to encourage the TS to complete his suicide plans. In contrast, three participants in seven different posts are more discouraging and try to talk him out of his suicide plans, for example:

‘Can't you tell us a little about your life TS?’

‘Don't do it, there are other solutions.’

It seems that the TS is affected by these more supportive posts, but at the same time not wanting to let go of his suicide plan:

‘Starting to feel that I'm about to change my mind about killing myself, so I have to hurry up a bit … ’

The TS does not give any clear explanation about why he wants to kill himself. In just one post he momentarily discusses what could be the reason:

‘I have Asperger syndrome/high-functioning autism. Am overly vulnerable (emotionally) … Have rather poor social skills, which makes me a somewhat lonely person.’

What the TS says is that his sensitivity, vulnerability and poor social skills make him lack social relations, and he is therefore a lonely person. The TS's only comment on why he chooses to live-stream his suicidal act is as follows:

‘Don't really know, have always felt that I want to broadcast my suicide ha-ha.’

In his last comment, just before he takes his own life, the TS writes:

‘alright lets do it.’

### During and after the suicide

#### Aspects of authenticity

A question raised in many posts in the thread is about authenticity, i.e. is this for real; is he really going to take his own life; is he just faking the situation to attract attention? The first comment the TS received after he posted his last words ‘alright let's do it’ was:

‘You're a troll, I think.’

Besides the aforementioned problems involved in suicide communication, people often distrust the authenticity of what others communicate on forums on the internet, especially when the participants can be anonymous and the communication is about more exceptional issues. Despite the fact that the TS did live-stream his suicide on the Flashback forum, 10% of the participants that posted on the issue (*n* = 470) during and after the suicide still believed that the suicide act was a fake.

#### Attitudes to the TS's suicide

**Fig. 2:**
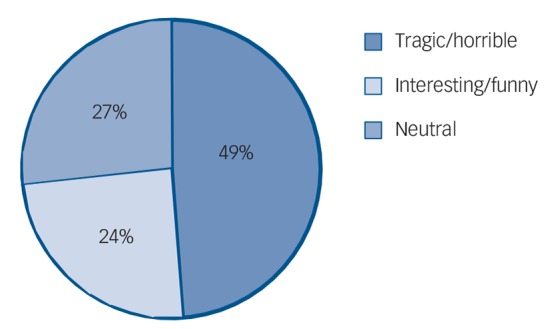
Attitudes to the thread starter's suicide (*n* = 344).

Of the 344 posted messages that express an opinion about the TS's suicide, nearly half (49%) state that his suicide is tragic, terrible or shocking (Fig. 2), for example:

‘Horrible. Moreover, this kind tends to rub off and inspire others. Hope that the TS is in a better place now.’

‘rest in peace TS. I was too late to write something that would make you change your mind. Very sad way to end one's life.’

‘Goddamn shit, so fucking tragic.’

However, 24% of the posts say that the suicide is exciting, interesting or funny (Fig. 2):

‘Call me sick, but have never laughed so much in my life lol [a common acronym used in internet slang that means “laughing out loud”].’

‘Well who cares … one tramp less, moreover, it is probably a fake.’

#### Opportunities for prevention

**Fig. 3:**
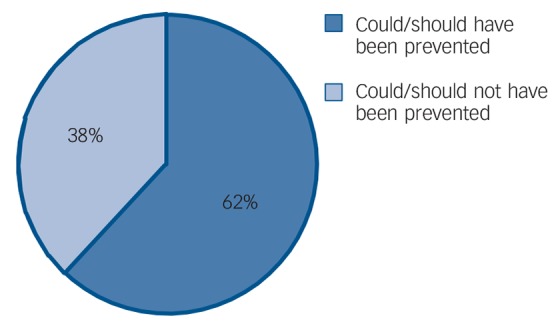
Opportunities for prevention (*n* = 95).

When it comes to beliefs and opinions about whether the suicide could have been prevented, a majority (62%, *n* = 59) of the posts on the issue imply that interacting with the TS could have stopped it (Fig. 3), for instance:

‘However, we could have acted faster. He could have survived if we had called him.’

‘I could have prevented it if I had called right away when he started the thread, but I did not?’

‘All these trolls on the internet have made it difficult to believe in threads like this.’

In contrast, a quite large proportion of the posts (38%, *n* = 36) indicate that you cannot, or should not, stop or interfere in other peoples suicide plans or acts (Fig. 3):

‘Ha-ha, awesome, if you want to kill yourself it's your own decision, no one should interfere.’

‘This is sick, but as I said. Why stop the guy? If he doesn't feel like living any longer, it is up to him to make the decision whether to do it or not.’

In this context it is worth noting that the moderator of the thread expressed the view that one should not interfere in other people's suicide plans or acts of suicide:

‘There are many reasons to commit suicide but I respect people who want it, after all it is their life and body, and I think they should be allowed to do what they want with those things.’

#### Responsibility for the TS's suicide

**Fig. 4:**
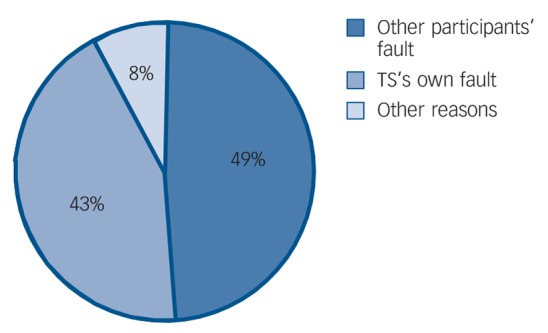
Responsibility for the thread starter's suicide (*n* = 110).

Almost half of the posts (49%) that discuss the question of responsibility state that it was the other participants' fault that the TS died, either by directly inciting him to take his own life or by being too passive in the conversation (Fig. 4), for example:

‘All these disgusting idiots on Flashback who incited him to do it. Hope you will suffer for the rest of your lives. Filthy bastards!’

‘I think that you should be so ashamed, you who wrote “good luck” “You will never dare,” etc., the fact that you can write something like that to a guy who obviously does not feel well is completely hellish, it could be those words that gave him the motivation to pursue it. Frankly, you have been involved in this.’

‘Sincerely hope that you carry this with you for the rest of your lives. You urged a guy to kill himself, which he did. The most tragic I've seen on Flashback. Are you satisfied now, fucking idiots!’

#### Reasons why the TS took his own life

**Fig. 5:**
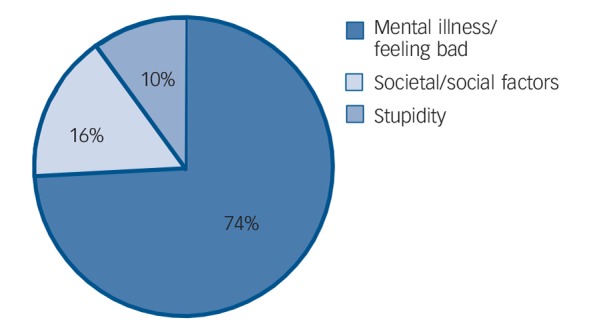
Reasons why the thread starter took his own life (*n* = 38).

Only 38 posts discuss possible reasons why the TS took his own life. Nearly three-quarters of these (74%, *n* = 28) claim that suicide is mainly about mental illness or that you are ‘feeling bad’ (Fig. 5):

‘The reason why he did it, I think, was probably due to autism and asperger, and his complex thoughts about the universe and life that made him curious about life after death.’

‘I really hope that you understand now, understand that these people who feel so bad that they can't see another way out.’

Another 16% (*n* = 6) point to external societal or social factors and 10% (*n* = 4) think it is about ‘stupidity’ (Fig. 5):

‘Money is probably a major reason for suicide in the West where you do not value the small things in life, like seeing your family or friends daily. Do not think he was honest with “I have a good life,” maybe the guy did not want people to feel sorry for him, I think he had a crappy life.’

‘That you make a spectacle of it and almost mocking yourself before you kill yourself simply indicates that the person was feeble-minded and weak as an individual. He wanted attention, and he got his 5 minutes in the spotlight at the price of his life … seems like a bad deal to me.’

#### Perceptions of the images of the TS's suicide

**Fig. 6:**
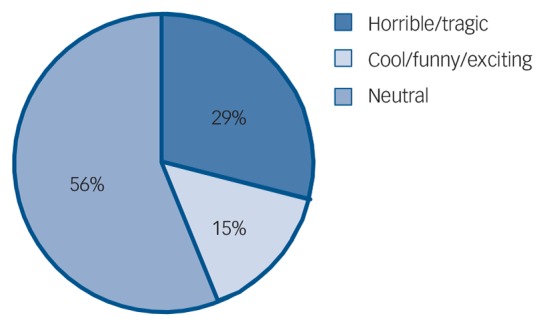
Perceptions of the images of the thread starter's suicide (*n* = 148).

Of the 148 posts that specifically comment on the broadcast of the TS's suicide, 29% express that it is horrible or tragic (Fig. 6), for example:

‘What has happened is truly tragic. I looked through all the pictures of the suicide and felt downright horrible after that.’

‘The guy was same fucking age as me, I also have Aspergers … hell, he even has the same TV table as I have. I see how people make images and yank him on 4chan [an image-based website], but I can not fucking laugh. The tears are running, poor guy.’

‘Have been hanging out on the internet most of my childhood and this was without a doubt the absolute worst I've ever seen in my entire cyber life. My thoughts go to him and all his relatives. RIP [rest in peace].’

However, 15% of the posts say that the images are exciting or funny (Fig. 6), such as:

‘Ha-ha-ha, this was a good day:P [:P is an emoticon frequently used on the internet to mean a smiley face, sticking out tongue.].’

‘Give me some more pictures please! Ideally, a video of all images, from that he started 'til that the cops came and shut it down <3 [<3 is a sideways heart-shaped internet emoticon to denote “love”].’

‘High-level fucking humour in all this. Especially I laughed at the image where the cops just discovered that they are being broadcast on the interwebs.’

The majority of the comments (56%) are more objective, or dispassionate, in their descriptions of the images (Fig. 6):

‘I read a post where someone claimed that he hung for 25 minutes? Is this true? There are a total of 223 images from the time he releases the body and restricts the oxygen supply to the police picking him up. According to him, the FTP updated every 2 seconds. So 223×2 = 446 seconds, 446 seconds = 7 minutes and 25 seconds’

## Discussion

In the present study, the internet forum Flashback can be seen as a (virtual) social environment, which, together with the young TS's communication and the other participants' different postings, constitute a specific suicide communication event.^20^ A suicide communication event begins with a speech act, or a message, from one person to another, and how this message is interpreted depends on the other participants' beliefs, knowledge and attitudes about suicide, as well as the communicative context.

### Before the suicide

The analysis of the postings before the suicide act showed that the TS was very straightforward and clear about his suicide intent and described previous violent suicidal behaviour. Testing and rehearsing suicide methods is a significant indicator that the individual has taken a further step in the suicide process. Joiner^27^ postulates that besides psychological states like ‘failed belongingness’ and ‘perceived burdensomeness,’ taking one's own life also requires the ‘ability to enact lethal self-injury’. Increased exposure to violent incidents and situations (like self-strangulation) can lead to the individual's instinctive fear of death diminishing or completely disappearing. Overall, this could be seen as an ‘alarming conversation’^28^ and should be taken as a serious suicide message, yet almost half of the posts following his declaration were either encouraging him to take his life, or doubting the authenticity of his posts. However, the analysis also shows that the supportive posts may have had a positive effect on the TS and that he was about to reconsider his decision. But at the same time he did not want to let go of his suicide plan.

This kind of ambivalence (‘to be or not to be’) is characteristic of the suicide process and can act as a protective factor.^29,30^ To hesitate before a suicide act means that the choice between life and death is an open question until the very end. This means that there is time to try to help and support the suicidal individual to stay alive, which the empathetic comments in the posts are examples of. Conversely, to incite or distrust a suicidal person in a communicative situation – which we also saw significant examples of in the thread – could shorten the ambivalence period and be fatal.^31^ As long as the dialogue proceeds, the subject is involved in a form of negotiation, both with themselves and with others about where they are headed. The conversation, as it were, keeps future alternatives relatively open. However, when the dialogue is interrupted or cannot be established, there is a risk that acts of suicide will be carried out. The acts become replacements for the absence of dialogue.^32^

The analysis also showed that the TS's reason for dying by suicide was essentially about social isolation and being lonely. In a study of intimate conversations on a suicide forum on the internet, loneliness in particular is singled out by many participants as a major cause of suicidal thoughts, plans and acts.^8^ This is often formulated in terms of being abandoned, not being seen or heard and that no one cares. Suffering pain, grief, anxiety and self-loathing, without being able to connect with another human being and be given the opportunity to share this burden, becomes overwhelmingly difficult for many people. Also, in many other theoretical and empirical works on suicidality^33,37^ loneliness is often presented as the factor that provides the tipping point.

The above discussion may also shed some light on why the TS chooses to live-stream his suicide on the forum. The only comment the TS himself gives about this is: ‘Don't really know, have always felt that I want to broadcast my suicide ha-ha’. Of course there can be many reasons why an individual wants to take their own life in public. One can be about loneliness. Perhaps the knowledge that other participants were watching the suicide gave the TS a sense of community, so he would not have to die alone. In another context, but still comparable, Ozawa-De Silva,^38,39^ points to the role of sociality in internet-based suicide pacts: in meeting, planning and carrying out suicide plans together, people can experience a sense of relationship and community. For those trying to establish suicide pacts on the internet, dying together seems more comforting than dying alone. When the TS chooses to ‘share’ his suicide with the other participants, it can be interpreted as a final attempt to break the social isolation and loneliness, although it may seem contradictory.

A suicide is not just about dying. A suicide also communicates something to the world around the person, such as that the person feels unloved and outcast. The suicidal person achieves something with the act, something that the recipients cannot escape. It is a way to regulate the social environment.^40^ The suicidal act can be seen as a powerless person's weapon to influence the outside world in a way in which the recipients are deprived of the ability to speak back, which is a fundamental point.

### During and after the suicide

The analysis of the messages that were posted after the suicide shows that a majority of the posts state that it could have been possible to prevent the TS's suicide, if just more of the participants in the thread had been supportive and fewer had questioned that he was serious and incited him to complete his suicide. This points to the idea that there was some awareness among the participants about the importance of how to respond to a suicidal individual, at least after the suicide is completed. But also, many of the participants (38%) expressed views that you should not interfere in other people's suicide plans or intentions (Fig. 3). The kinds of attitudes and beliefs people hold regarding suicide play an important role in responding to a suicidal individual's communication, and attitudes towards suicidal people can be very negative.^19,41,42^ Furthermore, only in 38 of the 608 posts were there discussions about the possible reasons leading up to the TS's suicide (Fig. 5).

Even if people want to help, lay people are often hesitant when confronting a person in a suicidal crisis, because of a lack of knowledge and the fear, anger and anxiety that such communication acts can awaken.^19,41^ A suicide communication event can be a highly face-threatening situation, both for the suicidal person and other people involved in the communication.^20^ The jokes and the ironic comments made by the participants, and the fact that some did not take the TS's posts seriously, could partly be understood by the taboo and stigma that surround suicide. Also, popular myths about suicide, such as ‘people who talk about suicide don't do it’ and ‘asking about suicidal thoughts may create suicidal ideas’ may discourage lay intervention.^20^ This could be a problem on internet forums like Flashback where many participants are rather suspicious because of the high level of deceptive posts and trolling. In this environment inaccurate assumptions and myths can continue to flourish.

### Implications

From previous studies we know that attitudes play a significant role in the suicidal process^41,43^ and that training programmes targeted at modifying knowledge, attitudes and behaviours seem promising at increasing help-seeking and help-offering behaviours and even reducing suicide attempts.^44–48^ In view of the fact that the internet has developed as the main channel for suicide communication^10^ (for example, the thread with the TS and the other participants has had about 5 million views to date) and that individuals with high suicide risk prefer to communicate on internet forums instead of communicating face-to-face in the physical world,^18^ it is of great importance to find methods and to develop new digital tools for identifying suicidal individuals by the way they communicate.^10,49,50^ Also important is to disseminate basic knowledge about the suicidal process, to increase awareness of suicide signals and understanding about how to respond to individuals who communicate suicide intentions on different forums on the internet. The moderator stated that one should not interfere in other people's suicide plans or acts of suicide, which in turn may have deterred those with a willingness to help the victim from acting. Education and training for moderators of internet forums, or managers of other types of interactive websites, may save lives in the future.

Another important task may be to raise awareness among clinicians about the risks and, perhaps, advantages involved in suicidal patients' internet activities. One suggestion is to establish routines in which clinicians ask their patients about their use of the internet, and preferably guide patients towards preventive sites with therapeutic resources.^12^ Also, the work of clinicians taking part in online communication platforms, where patients/lay persons discuss mental health-related topics, may facilitate the development of cohesion between patients and clinicians,^51^ and increase understanding of the patient perspective. In light of the present analysis and other studies, the internet can be a facilitator of the suicidal process, but it can also be a venue where opportunities for prevention of suicide loom large.

### Limitations

A limitation of the present study is that it only examines suicide communication at a single time on one forum on the internet. However, on interactive internet forums where communication about suicide takes place, different voices and different views may be heard on this problematic, taboo subject. Further studies with this focus can expand our understanding and knowledge of this complex and challenging field.
